# A systematic review of how studies describe educational interventions for evidence-based practice: stage 1 of the development of a reporting guideline

**DOI:** 10.1186/1472-6920-14-152

**Published:** 2014-07-24

**Authors:** Anna C Phillips, Lucy K Lewis, Maureen P McEvoy, James Galipeau, Paul Glasziou, Marilyn Hammick, David Moher, Julie K Tilson, Marie T Williams

**Affiliations:** 1 School of Health Sciences, University of South Australia, GPO box 2471, Adelaide 5001, Australia; 2 Health and Use of Time Group (HUT), Sansom Institute for Health Research, University of South Australia, GPO Box 2471, Adelaide 5001, Australia; 3 International Centre for Allied Health Evidence (iCAHE), School of Health Sciences, University of South Australia, GPO box 2471, Adelaide 5001, Australia; 4 Ottawa Hospital Research Institute, Centre for Practice-Changing Research (CPRC), The Ottawa Hospital, 501 Smyth Rd, K1H 8 L6, Ottawa, Ontario, Canada; 5 Centre for Research in Evidence-Based Practice (CREBP), Bond University, University Drive, Robina, Queensland 4226, Australia; 6 Bournemouth University, Royal London House, Christchurch Road, Bournemouth, Dorset, UK; 7 Ottawa Hospital Research Institute, Clinical Epidemiology Program, Centre for Practice-Changing Research (CPCR), The Ottawa Hospital, 501 Smyth Rd, K1H 8 L6, Ottawa, Ontario, Canada; 8 University of Southern California, Division of Biokinesiology and Physical Therapy, 1540 Alcazar St, CHP155, Los Angeles 90089, USA; 9 School of Population Health, Nutritional Physiology Research Centre (NPRC), University of South Australia, GPO Box 2471, Adelaide, 5001, Australia

**Keywords:** Systematic review, Evidence-based practice, Educational intervention, Reporting guidelines

## Abstract

**Background:**

The aim of this systematic review was to identify which information is included when reporting educational interventions used to facilitate foundational skills and knowledge of evidence-based practice (EBP) training for health professionals. This systematic review comprised the first stage in the three stage development process for a reporting guideline for educational interventions for EBP.

**Methods:**

The review question was *‘What information has been reported when describing educational interventions targeting foundational evidence-based practice knowledge and skills?’*

MEDLINE, Academic Search Premier, ERIC, CINAHL, Scopus, Embase, Informit health, Cochrane Library and Web of Science databases were searched from inception until October - December 2011. Randomised and non-randomised controlled trials reporting original data on educational interventions specific to developing foundational knowledge and skills of evidence-based practice were included.

Studies were not appraised for methodological bias, however, reporting frequency and item commonality were compared between a random selection of studies included in the systematic review and a random selection of studies excluded as they were not controlled trials. Twenty-five data items were extracted by two independent reviewers (consistency > 90%).

**Results:**

Sixty-one studies met the inclusion criteria (n = 29 randomised, n = 32 non-randomised). The most consistently reported items were the learner’s stage of training, professional discipline and the evaluation methods used (100%). The least consistently reported items were the instructor(s) previous teaching experience (n = 8, 13%), and student effort outside face to face contact (n = 1, 2%).

**Conclusion:**

This systematic review demonstrates inconsistencies in describing educational interventions for EBP in randomised and non-randomised trials. To enable educational interventions to be replicable and comparable, improvements in the reporting for educational interventions for EBP are required. In the absence of a specific reporting guideline, there are a range of items which are reported with variable frequency. Identifying the important items for describing educational interventions for facilitating foundational knowledge and skills in EBP remains to be determined. The findings of this systematic review will be used to inform the next stage in the development of a reporting guideline for educational interventions for EBP.

## Background

Evidence-based practice (EBP) is accepted as an integral skill for health professionals and EBP training is included as an accreditation requirement in many health professions [1]. As EBP has gained global currency as a decision making paradigm, the frequency and number of studies exploring educational strategies for developing knowledge and skills in EBP has increased.

A recent systematic review identified over 170 published studies investigating educational interventions aimed at facilitating skills and knowledge of EBP [2]. Despite the continued investment of time, effort and resources in EBP education, best practice in EBP education remains unclear [3]. Inconsistent and incomplete reporting of information in educational interventions for EBP is common, thereby limiting the ability to compare, interpret and synthesise findings from these studies. Researchers undertaking systematic reviews in EBP education frequently identify the lack of detailed reporting of the educational interventions as an issue [2-7]. In 2003, Coomarasamy, Taylor & Khan [5] had difficulty determining the type and dose of the intervention due to the poor reporting in the included studies. A decade later, the problem persists, with Maggio et al. [6] and Ilic & Maloney [3] unable to draw conclusions about the effectiveness of the EBP educational interventions included in their systematic review due to the incomplete descriptions of the interventions. The consistent appeal from authors of systematic reviews is for improved detail in the reporting of educational interventions for EBP. The specific requests from authors of systematic reviews include improvements in the detail for the reporting of the development, implementation and content of the curriculum for the intervention, the employment of more rigorous study designs and methodology, and the use of robust outcome measures [2-7].

Reporting guidelines in the form of a checklist, flow diagram or explicit text provide a way for research reporting to be consistent and transparent [8]. The reporting guidelines specific to study design such as STROBE for observational studies [9], PRISMA for systematic reviews and meta-analysis [10] and CONSORT for randomised trials [11] have paved the way for greater accuracy in the reporting of health research [12]. The EQUATOR Network encourages high quality reporting of health research and currently includes some 218 reporting guidelines for different research approaches and designs [13].

There are four reporting guidelines currently listed on the EQUATOR Network website which are specific to educational interventions [14-17]. These include educational interventions in Cancer Pain education [15], Team Based Learning [16], Standardised Patients [14] and Objective Structured Clinical Examinations (OSCE) [17]. Other than the inclusion of a narrative literature review, the development processes used for these reporting guidelines differed and no formal consensus processes were reported for any of these reporting guidelines. The end user framework used for these reporting guidelines share some similarities. Howley et al. [14] and Patricio et al. [17] employ a checklist format, comprised of 18 [17] to 45 [14] items. Haidet et al. [16] and Stiles et al. [15] include a series of domains and recommendations for reporting in each domain. The information items included in each of these reporting guidelines are content specific. For example, Patricio et al. [17] include 31 items related specifically to the set up and design for OSCE’s. Howley et al. [14] include nine items specific to behavioural measures for standardised patients. None of the four reporting guidelines appeared to be appropriate for reporting educational interventions for developing knowledge and skills in EBP. Therefore an original three stage project was commenced, based on the recommendations for developers of reporting guidelines for health research [18], to develop the guideline for reporting evidence-based practice educational interventions and teaching (GREET) [19]. The aim of this systematic review was to identify which items have been included when reporting educational interventions used to facilitate foundational skills and knowledge for EBP. The data obtained from this review will be used to inform the development for the GREET [19].

The review question was: ‘What information has been reported when describing educational interventions targeting foundational evidence-based practice knowledge and skills?’

## Methods

### Research team

The research team consisted of a doctoral candidate (AP), experts with prior knowledge and experience in EBP educational theory (MPM, LKL, MTW), lead authors of the two Sicily statements (PG, JKT) and experts with experience in the development of reporting guidelines and the dissemination of scientific information (DM, JG, MH).

### Data sources

The search strategy underwent several iterations of peer-review before being finalised [20]. The search protocol was translated for each of the databases with four primary search themes, health professionals (e.g. medicine, nursing, allied health); EBP (e.g. EBM, best evidence medical education, critical appraisal, research evidence); education (e.g. teach, learn, journal club); and evaluation (e.g. questionnaire, survey, data collection) [19]. The preliminary search strategy was test run by two pairs of independent reviewers (AP and HB/MPM/JA) for each database. Inconsistencies between search results were discussed, the source of disagreement identified, and the searches were re-run until consistent. Between October and December 2011 the final search was completed of nine electronic databases (MEDLINE, Academic Search Premier, ERIC, CINAHL, Scopus, Embase, Informit health database, Cochrane Library and Web of Science). The MEDLINE search strategy is provided as an example in Additional file 1.

Protocols for systematic reviews are recommended to be prospectively registered where possible [10]. However, as this systematic review focussed on the reporting of educational interventions for EBP rather than a health related outcome, it was not eligible for prospective registration with databases such as PROSPERO [21].

#### Study selection

Eligibility criteria for studies are presented in Table 1. The reference lists of systematic reviews with or without meta-analysis identified in the search were also screened for further eligible studies.

**Table 1:** Study eligibility and exclusion criteria

**Inclusion criteria**	***Design***: randomised or non-randomised published in peer reviewed journals irrespective of language or date of publication.
	***Population***: health professionals > 18 years of age irrespective of the level of education of the learner (undergraduate, postgraduate), including medicine, nursing and allied health (physiotherapy, occupational therapy, speech therapy, dietetics, social work, psychology, podiatry ambulance paramedic, ambulance office, music therapy, art therapy, osteopathy, chiropractic, dentistry, optometry, medical radiations, pharmacy and exercise physiology professions) [22].
	***Intervention***: All types of educational interventions (e.g. journal club, workshop, short course) or education to facilitate the development of any of the five foundational steps of EBP (ask, acquire, appraise, apply and assess) [23].
	***Comparator***: All comparators: different educational intervention, ‘usual care’ or no intervention.
	***Outcomes***: Twenty-five potential data items in five prospectively determined domains developed using the CONSORT statement [11] and categories used by Robb et al. [24]: (1) Participants/Instructors (8 items), (2) Intervention mode and delivery (9 items), (3) Intervention content (3 items), (4) Evaluation (4 items) and (5) Confounding issues (1 item).
**Exclusion criteria**	Studies:
	• without a control group; or narratives, letters and books providing recommendations or strategies for teaching skills in EBP.
	• which described or reported evidence-based guidelines or educational interventions specific to health conditions rather than educational interventions to develop skills and knowledge of EBP (e.g. evidence-based education for conservative management of hip osteoarthritis).
	• which focused on educational interventions for facilitating learning of statistical concepts without addressing at least one of the five EBP steps.
	• that reported barriers, facilitators, attitudes, and behaviours relating to EBP without an educational intervention.

### Study selection and quality assessment

#### Training

A training exercise was undertaken to establish a consistent process for reviewing the title and abstracts against the eligibility criteria. Four reviewers (AP, MPM, LKL, MTW) collaboratively examined the title and abstracts of the first 150 citations for eligibility with disagreements resolved by consensus.

Once consistency was established, one investigator (AP) reviewed the titles and abstracts of the remaining citations. When titles and/or abstracts met the inclusion criteria or could not be confidently excluded, the full text was retrieved. The resultant list was reviewed by two independent reviewers (AP,MTW) for eligibility, with disagreements resolved by consensus. The reference lists of all included studies were screened, with 54 further potential citations identified. The penultimate list of eligible studies was reviewed (JKT, PG, MH, DM, JG) and three additional citations were nominated.

Eligibility was limited to controlled trials. To estimate whether reported items differed between controlled trials and lower level study designs, a random selection of 10 studies identified in the search using lower level study designs (pre-post studies without a separate control group) were compared with 15 randomly selected randomised and non-randomised trials (with control groups) for frequency and commonality of reporting items.

Studies were not appraised for methodological bias because the aim of this systematic review was to describe how EBP educational interventions have been reported rather than describing the efficacy of the interventions [25].

### Data extraction and treatment

A data extraction instrument was prospectively planned, developed and published [19] based on the Cochrane Handbook for Systematic Reviews of Interventions [26]. As outlined in the study protocol for the GREET, the 25 data items were extracted across domains including Participants, Intervention, Content, Evaluation and Confounding (Table 2). All data items were initially recorded verbatim. Consistency between extractors (AP, MW, LKL, MPM) was confirmed using a random sample of 10 per cent of eligible studies (inter-rater consistency >90% agreement). Data extraction was then completed by pairs of independent reviewers (AP and either LKL, MTW, MPM) and disagreements were resolved by discussion to reach consensus.

**Table 2:** Data extraction domains and information items

	**Data extracted**
**Participants***Learners’*	Context* of education/stage of training, number and type of professional discipline, previous EBP exposure, adherence/attendance.
*Instructors’*	Number involved, profession, previous teaching experience.
**Intervention**	Type of intervention, strategies for teaching/learning, educational framework.
	Number, frequency, duration, mode of delivery, materials provided and setting for learning sessions.
	Duration of program learning program, statement of student time not face-to-face.
**Content**	Steps of EBP covered in intervention, reference for EBP content, EBP steps described e.g. (ask, acquire, appraise, apply and assess).
**Evaluation**	Name and type of assessment method (e.g. exam, assignment, outcome tool), whether same evaluation method used for all groups, psychometric properties, whether a named test was used and/or modified.
**Confounding issues**	Verbatim statements of issues confounding the EBP educational intervention or interpretation of the learning outcome.

*The context of education refers to the year level of the learner within an undergraduate or post-graduate degree or course.

The 25 data items were grouped according to the frequency of reporting (ranging from low to very high frequency of reporting) and further reviewed to determine their role in relation to the reporting of the intervention. To provide an objective guide for differentiating between information items relating specifically to the intervention and those relating to the reporting of study design/methodology two reporting guidelines were used. The Template for Intervention Description and Replication (TIDIER) [27] was used to identify information items considered to be specific to the reporting of the intervention, and the CONSORT statement (excluding item 5, intervention) was used to identify information items which were considered to be related to the study design/methodology and confounding issues [11].

## Results

### Characteristics of eligible studies

**Figure 1:**
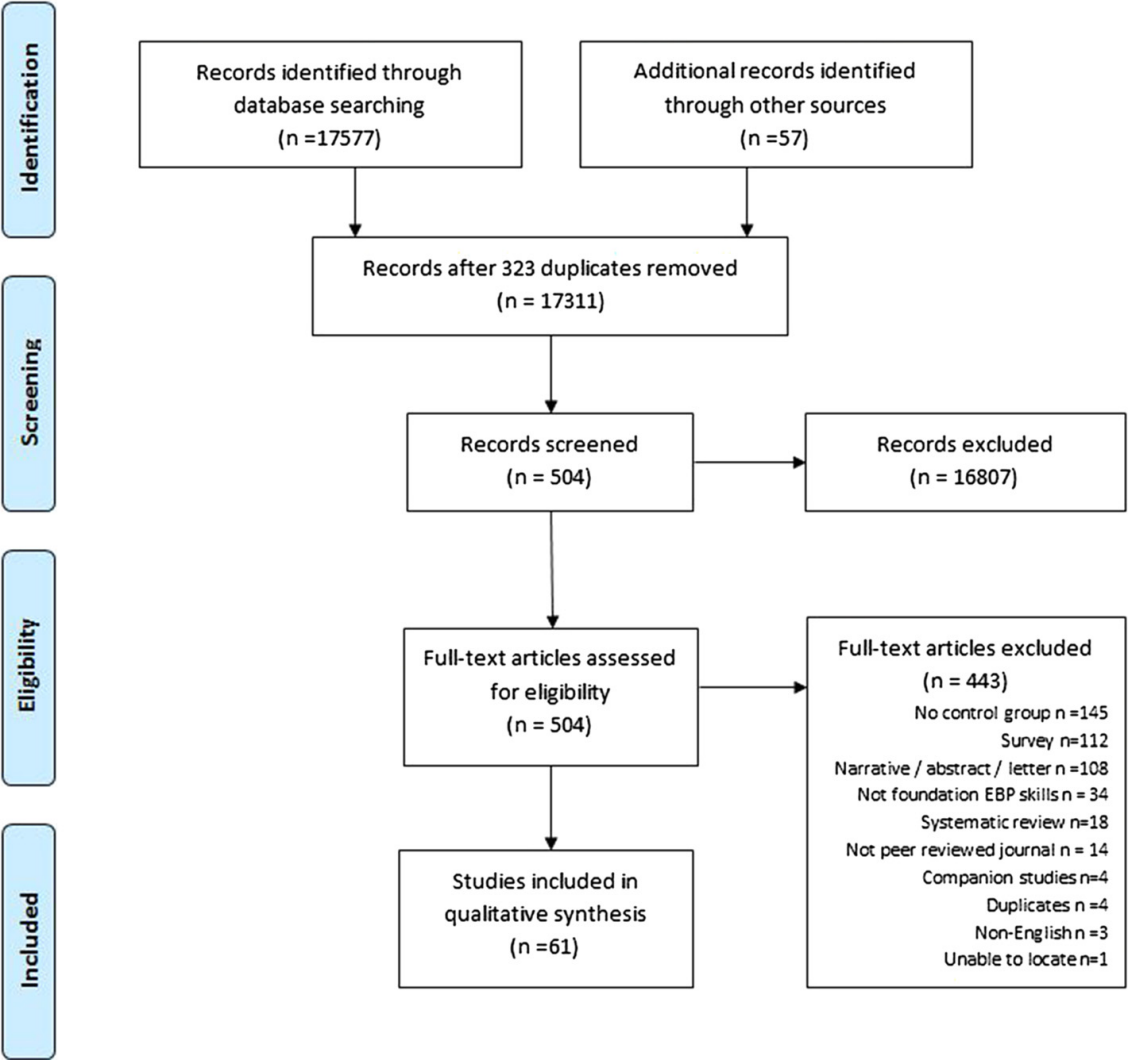
PRISMA flow diagram.

**Figure 2:**
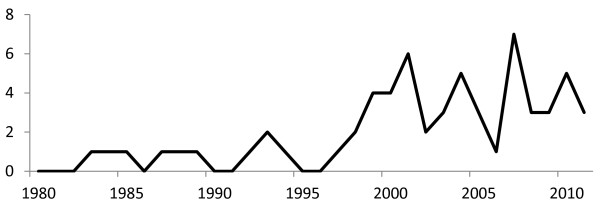
Publication frequency for studies included in systematic review.

Sixty-one studies met the inclusion criteria [4,28-87] (Figure 1) with all of these published in English. The median publication year was 2003 (range 1984 to 2011) with increasing frequency after 2000 (Figure 2). Studies were published in 34 journals with the most frequent being the Journal of General Internal Medicine (n = 7, 11%), BioMedical Central Medical Education (n = 6, 10%), Academic Medicine (n = 4, 7%) and Medical Education (n = 4, 7%).

There were approximately equal numbers of randomised (n = 29, 48%) and non-randomised (n = 32, 52%) trials. Two studies referenced the use of a reporting guideline [36,85] with both studies using the 2001 CONSORT statement [88].

### Frequency of reporting

The frequency of reporting of the 25 items across the five domains was evaluated for all studies (Additional file 2). Frequency of reporting, was described as very high (reported by ≥90% of studies), high (70-89%), moderate (50-69%) and low (<50%) (Table 3).

**Table 3:** Summary of the reporting of the data items for included studies (n = 25)

**Domain**					**Information item**	**No. studies reporting (%)**	**Reporting frequency**
**Participants**	**Delivery**	**Content**	**Evaluation**	**Confounding**			
**x**					Context of education/stage of training	61 (100)	Very high
**x**					Professional discipline	61 (100)	
**x**					Number of different disciplines	61 (100)	
			**x**		Evaluation method	61 (100)	
	**x**				Strategies for teaching/learning	59 (97)	
				**x**	Confounding issues/study limitations	57 (93)	
			**x**		Same evaluation method for all groups	57 (93)	
	**x**				Number of teaching sessions	51 (84)	High
	**x**				Duration of program	50 (82)	
	**x**				Setting	49 (80)	
		**x**			EBP steps covered in intervention content	46 (75)	
	**x**				Educational materials used in intervention	45 (74)	
	**x**				Frequency of sessions	44 (72)	
	**x**				Duration of sessions	42 (69)	Moderate
			**x**		Psychometric properties of evaluation method reported	35 (57)	
**x**					Previous EBP/research training exposure	30 (49)	Low
**x**					Instructors profession	27 (44)	
**x**					Number of instructors involved	24 (39)	
**x**					Learners adherence/attendance	24 (39)	
	**x**				Educational framework	22 (36)	
		**x**			Reference for EBP content reported	18 (30)	
			**x**		Evaluation instrument name and if modified	12 (21)	
		**x**			Reference to term ‘steps of EBP’ for content of intervention	9 (15)	
**x**					Instructors previous teaching experience	8 (13)	
	**x**				Statement of student time not face to face	1 (2)	
8	9	3	4	1			Total

EBP Evidence-based practice.

#### Information items reported with very high frequency (≥90% of studies)

Seven items were reported with very high frequency (Table 3). Four items from two domains were reported by all studies (Participant domain: *context of education/stage of training, professional discipline, number of disciplines*; Evaluation domain: *evaluation methods*). The remaining three items included the *strategies used for teaching and learning* (Intervention domain: n = 59, 97%); *whether the same evaluation method was used for all groups* (Evaluation domain: n = 57, 93%); and *confounding issues or study limitations* (Confounding issues domain: n = 57, 93%).

#### Information items reported with high frequency (70 to 89% of studies)

Six items were reported with high frequency (Table 3). The majority were from the Intervention domain including the *number of sessions* (n = 51, 84%), *program duration* (n = 50, 82%), *setting* (n = 49, 80%), *frequency of the sessions* (n = 44, 72%) and the *educational materials used* (n = 45, 74%). The remaining items reflected which EBP steps were included in the intervention (Content domain n = 46, 75%). The most frequently reported EBP step was Step 3 (appraise) (n = 46, 75%) followed by Step 2 (acquire) (n = 38, 62%) and Step 1 (ask) (n = 30, 49%). The two least frequently reported were Steps 4 (apply) (n = 23, 38%) and 5 (assess) (n = 4, 7%).

#### Information items reported with moderate frequency (50 to 69% of studies)

Two items were reported with moderate frequency: *duration of sessions* (Intervention domain: n = 42, 69%) and *psychometric properties of the evaluation method* (e.g. face validity, inter-rater reliability) [89] (Evaluation domain: n = 35, 57%).

#### Information items reported with low frequency (<50% of studies)

There were ten information items which were reported with low frequency (Table 3). Half of the items were from the Participants/Instructors domain including *previous EBP or research training exposure of the learners* (n = 30, 49%), *adherence or attendance at the intervention* (n = 24, 39%), *profession of the instructors* (n = 2, 44%), *number of instructors involved* (n = 24, 39%) and *previous teaching experience* (n = 8, 13%). Two items were from the Intervention domain (*educational framework* n = 22, 36% and *student time spent not face to face* n = 1, 2%) and two items were from the Evaluation domain *(citation provided for EBP content)* n = 18, 30% and *citation provided for steps of EBP for the content of the intervention* n = 9, 15%). The remaining item concerned whether the *name of the evaluation instrument* was identified and *whether it was modified* (n = 12, 21%) (Evaluation domain).

For the studies that provided a citation to describe the steps or components of EBP (n = 18, 30%), the most commonly reported were Sackett et al. (Evidence-Based Medicine: How to Practice and Teach EBM) [90] (n = 12,67%) and the Evidence-Based Medicine Working Group [91] (n = 5,26%).

#### Items relating specifically to the reporting of the intervention

When the 25 data extraction items were sorted into those related to the reporting of interventions (TIDIER) [27] and items related to study design (CONSORT) [11], most of the items (n = 16, 64%) were considered to be specific to the reporting of intervention rather than the study design (n = 9, 36%) (Table 4).

**Table 4:** Items specific to the reporting of interventions (n = 16) allocated to the TIDIER framework

**Domain**				**Information item**	**No. studies reporting (%)**
**Instructors**	**Delivery**	**Content**	**Confounding**		
	**x**			Strategies for teaching/learning	59 (97)
			**x**	Confounding issues/study limitations relating to intervention	57 (93)
	**x**			Number of teaching sessions	51 (84)
	**x**			Duration of program	50 (82)
	**x**			Setting	49 (80)
		**x**		EBP steps covered in intervention content	46 (75)
	**x**			Educational materials used in intervention	45 (74)
	**x**			Frequency of sessions	44 (72)
	**x**			Duration of sessions	42 (69)
**x**				Instructors profession	27 (44)
**x**				Number of instructors involved	24 (39)
**x**				Learners adherence/attendance	24 (39)
	**x**			Educational framework	22 (36)
		**x**		Reference for EBP content reported	18 (30)
		**x**		Reference to term ‘steps of EBP’ for content of intervention	9 (15)
**x**				Instructors previous teaching experience	8 (13)

#### Confounding

There were 197 issues reported by authors of 57 (93%) studies as either limitations or factors which may have confounded the results of the educational intervention (Additional file 3). There was little commonality across the confounding items relating to the intervention, with almost one quarter of the studies (n = 10, 24%) reporting limitations relating to the delivery, duration or time of year for the educational intervention program.

## Discussion

The aim of this systematic review was to determine what information is reported in studies describing educational interventions used to facilitate foundational skills and knowledge for EBP.

Stiles et al. [15] use the term ‘educational dose’ to describe information such as the duration of the educational intervention, learning environment, the extent and intensity of direct interactions with the educators and the extent of the institution support. This educational dose is considered a core principle of an educational intervention [15]. For an educational intervention for EBP to be replicated, compared or synthesised, a detailed description of this educational dose is essential. However, this current review and several previous reviews [2-7], have identified inconsistent reporting of information items for the educational dose in studies of educational interventions.

The most consistently reported items across the 61 included studies were the *learners context of education/stage of training, professional discipline of the learners, number of different disciplines of the learners and the evaluation method used*, which were reported by all of the included studies. The most consistently reported domain was the Intervention delivery, with six out of nine items (67%) reported by more than 70 per cent of studies.

Comparison of the most consistently reported items in this review to other reviews of educational interventions undertaken as part of the development for a reporting guideline, reveals similar results. The learners’ stage of education was found to be reported by 97 per cent [14] and 83.8 per cent [17] of studies. The professional discipline and number of different disciplines of the learners was not reported for any of these systematic reviews of educational interventions. It is possible that this is due to these previous reviews being based solely on the medical profession. The evaluation method used, reported by all studies in this systematic review, was reported by between 25 [14] and 67.8 per cent [16] of studies.

The least consistently reported domain in our study was the ‘participants/instructors’ due to the limited reporting of detail regarding the instructor(s). Information regarding the number of instructors and their professional discipline and teaching experience was often not reported. These results are not unique to our findings. Maggio et al. [6] were not able to determine the instructors profession in 40 per cent of studies. Patricio et al. [17] found information regarding the number and detail of the faculty involved in the intervention was missing for 85.7% of studies and Haidet et al. [16] found no information regarding the faculty background in Team Based Learning reported for any of the included studies.

While every effort was made to plan and undertake a comprehensive search strategy, there are several potential limitations for this review. This systematic review was undertaken using the PRISMA reporting guideline [10] which includes recommendations for a number of strategies to identify sources of potential eligible articles. The screening of citations of included articles (progeny) is not currently included in the PRISMA reporting guideline, and was not undertaken as part of this review. However, in theory, if a review of progeny were included, relevant existing studies (similar topic, within search strategy, within included databases and within timeframe) should have been identified by the original search strategy.

The final search terms included the health professional disciplines of medicine, nursing and allied health; allied health disciplines included were based on the definition by Turnbull et al. [22]. It is possible that some professions, such as complimentary medicine, could have been missed. However, this risk was minimised by using a search string that included all relevant terms pertaining to EBP (e.g. evidence-based practice; evidence based medicine; EBM; best evidence medical education; BEME; research evidence). All studies, irrespective of professional discipline, should have been identified. During the initial search phase we did not apply language limits, however while reviewing the final list of eligible studies the decision was made to exclude three studies published in Spanish. It is unlikely that exclusion of these studies, which accounted for approximately four per cent of the eligible studies, would meaningfully alter our results.

Despite the development and testing of a prospective data extraction process, the allocation of items into pre-determined domains had the potential to overlook important information items and introduce bias. This systematic review was planned as the first of a three stage development process for the GREET. The purpose of the systematic review (stage 1) was to determine what had previously been reported in educational interventions for EBP to inform the second stage of the development process, the Delphi survey. The Delphi survey was planned to seek the prospective views of experts in EBP education and research regarding which information should be reported when describing an intervention to facilitate knowledge and skills in EBP. In order to ensure that the widest possible range of items were considered in the third stage of the reporting guideline development process, it was prospectively planned that all items identified within the systematic review would be included for comment in the Delphi process.

Finally, it is not always possible or practical to have a control group in studies investigating the effectiveness of educational interventions, hence the findings from this review may be limited by the exclusion of lower level study designs. Although our analysis comparing a small number of included studies to studies excluded based on design suggest that the reporting of educational interventions for EBP is similar irrespective of research design.

The usefulness of reporting guidelines for research designs such as systematic reviews (PRISMA) [10] and randomised controlled trials (CONSORT) [11] is well established, with many leading journals and editors endorsing these guidelines. These guideline documents are dynamic; the CONSORT checklist is continually updated as new evidence emerges. For example, selective outcome reporting was added to the 2010 CONSORT update [11]. In reporting guidelines for study designs, there is usually an item relating to the reporting of an intervention in addition to items relating to study methodology and analysis. In CONSORT, one item pertains to the reporting of the intervention (item 5: reporting of the interventions for each group with sufficient details to allow replication, including how and when they were actually administered). Given the number of information items identified within this systematic review, we believe that authors will benefit from guidance regarding the detail necessary to support replication and synthesis of educational interventions for EBP.

There are extensions to CONSORT for the reporting of interventions such as herbal and homeopathic interventions [92,93], non-pharmacological treatments [94], acupuncture [95], E-Health [96] and tailored interventions [97]. The collaborators of the CONSORT group have recently developed the TIDIER checklist and guide [27], a generic reporting guideline for interventions, irrespective of the type of intervention, where no other specific guidance exists. Although there are four reporting guidelines for educational interventions, none of these have been developed using a formal consensus process, nor do they relate specifically to educational interventions for EBP. The findings of this review suggest that there is need for supplemental reporting guidelines (to expand the single item in CONSORT) to address the reporting of educational interventions for EBP.

The determination of which items are necessary for describing an educational intervention is a complex task. Empirical evidence for which items are likely to introduce bias in educational interventions for EBP is scarce, largely due to the inconsistent and incomplete reporting for studies reporting educational interventions for EBP [2-7]. Information reported by authors as confounders or limitations may provide anecdotal evidence regarding which information items may introduce bias or impact upon study outcomes. The most frequently reported limitations by the authors related to the delivery, duration or the time of year for the educational intervention (n = 10, 24%).

## Conclusion

This systematic review collated information concerning what has been reported in the description of educational interventions for EBP. Completing the first stage in the development process for a reporting guideline specific for educational interventions for EBP (GREET) [19], the findings of this review provide a starting point for a discussion regarding the types of items that should be included in the GREET. The second stage in the development process for the GREET, a Delphi consensus survey, will be informed by the findings of this review. The GREET will be the first intervention-specific reporting guideline, based on the TIDIER framework, and will provide specific guidance for authors of studies reporting educational interventions for EBP.

## Competing interests

Dr. Moher is supported by a University Research Chair. Dr. Moher is a member of the EQUATOR executive committee.

## Authors’ contributions

AP, LKL, MPM, MTW, planned and undertook the systematic review, analysed the results and completed the first draft of the manuscript. PG, DM, JG, JKT, MH, contributed to systematic review protocol development, analysis of the results and drafting of the manuscript. All authors read and approved the final manuscript.

## Authors’ information

AP is a PhD candidate, School of Health Sciences, University of South Australia, Adelaide, Australia.

LKL is a Post-doctoral Research Fellow, Health and Use of Time Group (HUT), Sansom Institute for Health Research, School of Health Sciences, University of South Australia, Adelaide, Australia.

MPM is a Lecturer, School of Health Sciences and a member of the International Centre for Allied Health Evidence (iCAHE), University of South Australia, Adelaide, Australia.

JG is a Senior Research Associate, Ottawa Hospital Research Institute, The Ottawa Hospital, Centre for Practice-Changing Research (CPCR), Ontario, Canada.

PG is the Director, Centre for Research in Evidence-BasedPractice (CREBP),Bond University, Queensland, Australia.

DM is a Senior Scientist, Clinical Epidemiology Program, Ottawa Hospital Research Institute, The Ottawa Hospital, Centre for Practice-Changing Research (CPCR), Ontario, Canada.

JKT is an Associate Professor, University of Southern California Division of Biokinesiology and Physical Therapy, Los Angeles, USA.

MH is a visiting Professor, Bournemouth University, Bournemouth, UK and a consultant to Best Evidence Medical Education (BEME).

MTW is an Associate Professor, School of Population Health and a member of the Nutritional Physiology Research Centre (NPRC), School of Health Sciences, University of South Australia, Adelaide, Australia.

Correspondence should be addressed to Ms Anna Phillips, School of Health Sciences, University of South Australia, GPO box 2471, Adelaide 5001, Australia.

## Pre-publication history

The pre-publication history for this paper can be accessed here:

http://www.biomedcentral.com/1472-6920/14/152/prepub

## Supplementary Material

Additional file 1 **MEDLINE Search strategy for the OVID interface.** The MEDLINE search strategy we used for the systematic review using the OVID interface. Click here for file

Additional file 2 **Summary of data items reported for included studies.** A summary of the reporting for the 25 data extraction items across the studies (n = 61) included in the review. Click here for file

Additional file 3 **Confounding items/study limitations allocated using the CONSORT checklist.** A summary table of the confounding information items and study limitations reported by included studies which were allocated using the CONSORT checklist items. Click here for file

## References

[B1] Tilson JK Kaplan SL Harris JL Hutchinson A Ilic D Niederman R Potomkova J Zwolsman SE Sicily statement on classification and development of evidence-based practice learning assessment tools BMC Med Educ 2011 11 78 21970731 10.1186/1472-6920-11-78 PMC3221624

[B2] Young T Rohwer A Volmink J Clarke M What Are the Effects of Teaching Evidence-Based Health Care (EBHC)? Overview of Systematic Reviews PLoS ONE 2014 9 1 e86706 24489771 10.1371/journal.pone.0086706 PMC3904944

[B3] Ilic D Maloney S Methods of teaching medical trainees evidence-based medicine: a systematic review Med Educ 2014 48 2 124 135 24528395 10.1111/medu.12288

[B4] Fritsche L Neumayer H Kunz R Greenhalgh T Falck-Ytter Y Do short courses in evidence based medicine improve knowledge and skills? BMJ 2002 325 7376 1338 12468485 10.1136/bmj.325.7376.1338 PMC137813

[B5] Coomarasamy A Taylor R Khan K A systematic review of postgraduate teaching in evidence-based medicine and critical appraisal Med Teach 2003 25 1 77 81 14741863 10.1080/0142159021000061468

[B6] Maggio LA Tannery NH Chen HC ten Cate O O'Brien B Evidence-based medicine training in undergraduate medical education: a review and critique of the literature published 2006–2011 Acad Med 2013 88 7 1022 1028 23702528 10.1097/ACM.0b013e3182951959

[B7] Wong SC McEvoy MP Wiles LK Lewis LK Magnitude of change in outcomes following entry-level evidence-based practice training: a systematic review Int J Med Educ 2013 4 107 114

[B8] Simera I Altman DG Moher D Schulz KF Hoey J Guidelines for reporting health research: the EQUATOR network's survey of guideline authors PLoS Med 2008 5 6 e139 18578566 10.1371/journal.pmed.0050139 PMC2443184

[B9] von Elm E Altman DG Egger M Pocock SJ Gotzsche PC Vandenbroucke JP STROBE Initiative The Strengthening the Reporting of Observational Studies in Epidemiology (STROBE) statement: guidelines for reporting observational studies Epidemiology 2007 18 6 800 804 18049194 10.1097/EDE.0b013e3181577654

[B10] Moher D Liberati A Tetzlaff J Altman DG PRISMA Group Preferred reporting items for systematic reviews and meta-analyses: the PRISMA statement PLoS Med 2009 6 7 e1000097 19621072 10.1371/journal.pmed.1000097 PMC2707599

[B11] Schulz KF Altman DG Moher D CONSORT Group CONSORT 2010 Statement: updated guidelines for reporting parallel group randomised trials BMC Med 2010 8 18 20619135 10.3736/jcim20100702

[B12] Turner L Shamseer L Altman DG Weeks L Peters J Kober T Dias S Schulz KF Plint AC Moher D Consolidated standards of reporting trials (CONSORT) and the completeness of reporting of randomised controlled trials (RCTs) published in medical journals Cochrane Database Syst Rev 2012 11 MR000030 10.1002/14651858.MR000030.pub2 PMC7386818 23152285

[B13] The EQUATOR network [http://www.equator-network.org/]

[B14] Howley L Szauter K Perkowski L Clifton M McNaughton N Association of Standardized Patient Educators (ASPE) Quality of standardised patient research reports in the medical education literature: review and recommendations Med Educ 2008 42 4 350 358 18298448 10.1111/j.1365-2923.2007.02999.x

[B15] Stiles CR Biondo PD Cummings G Hagen NA Clinical trials focusing on cancer pain educational interventions: core components to include during planning and reporting J Pain Symptom Manage 2010 40 2 301 308 20541899 10.1016/j.jpainsymman.2009.12.011

[B16] Haidet P Levine RE Parmelee DX Crow S Kennedy F Kelly PA Perkowski L Michaelsen L Richards BF Perspective: guidelines for reporting team-based learning activities in the medical and health sciences education literature Acad Med 2012 87 3 292 299 22373620 10.1097/ACM.0b013e318244759e

[B17] Patricio M Juliao M Fareleira F Young M Norman G Vaz Carneiro A A comprehensive checklist for reporting the use of OSCEs Med Teach 2009 31 2 112 124 19330670 10.1080/01421590802578277

[B18] Moher D Schulz KF Simera I Altman DG Guidance for developers of health research reporting guidelines PLoS Med 2010 7 2 e1000217 20169112 10.1371/journal.pmed.1000217 PMC2821895

[B19] Phillips AC Lewis LK McEvoy MP Galipeau J Glasziou P Hammick M Moher D Tilson J Williams MT Protocol for development of the guideline for reporting evidence based practice educational interventions and teaching (GREET) statement BMC Med Educ 2013 13 9 23347417 10.1186/1472-6920-13-9 PMC3599902

[B20] Sampson M McGowan J Cogo E Grimshaw J Moher D Lefebvre C An evidence-based practice guideline for the peer review of electronic search strategies J Clin Epidemiol 2009 62 9 944 952 19230612 10.1016/j.jclinepi.2008.10.012

[B21] The PROSPERO database [http://www.crd.york.ac.uk/PROSPERO/]

[B22] Turnbull C Grimmer-Somers K Kumar S May E Law D Ashworth E Allied, scientific and complementary health professionals: a new model for Australian allied health Aust Health Rev 2009 33 1 27 37 19203331 10.1071/ah090027

[B23] Dawes M Summerskill W Glasziou P Cartabellotta A Martin J Hopayian K Porzsolt F Burls A Osborne J Second International Conference of Evidence-Based Health Care Teachers and Developers Sicily statement on evidence-based practice BMC Med Educ 2005 5 1 1 15634359 10.1186/1472-6920-5-1 PMC544887

[B24] Robb SL Burns DS Carpenter JS Reporting guidelines for music-based interventions J Health Psychol 2011 16 2 342 352 20709884 10.1177/1359105310374781 PMC3141224

[B25] Moher D Weeks L Ocampo M Seely D Sampson M Altman DG Schulz KF Miller D Simera I Grimshaw J Hoey J Describing reporting guidelines for health research: a systematic review J Clin Epidemiol 2011 64 7 718 742 21216130 10.1016/j.jclinepi.2010.09.013

[B26] Higgins JPT Green S The Cochrane Collaboration Cochrane Handbook for Systematic Reviews of Interventions 2009 Version 5.0.2

[B27] Hoffmann TC Glasziou PP Boutron I Milne R Perera R Moher D Altman DG Barbour V Macdonald H Johnston M Lamb SE Dixon-Woods M McCulloch P Wyatt JC Chan A Michie S Better reporting of interventions: template for intervention description and replication (TIDieR) checklist and guide BMJ 2014 348 1687 10.1136/bmj.g1687 24609605

[B28] Akl EA Izuchukwu IS El-Dika S Fritsche L Kunz R Schunemann HJ Integrating an evidence-based medicine rotation into an internal medicine residency program Acad Med 2004 79 9 897 904 15326018 10.1097/00001888-200409000-00018

[B29] Arlt SP Heuwieser W Training students to appraise the quality of scientific literature J Vet Med Educ 2011 38 2 135 140 22023921 10.3138/jvme.38.2.135

[B30] Badgett RG Paukert JL Levy LS Teaching clinical informatics to third-year medical students: negative results from two controlled trials BMC Med Educ 2001 1 3 11532204 10.1186/1472-6920-1-3 PMC48153

[B31] Bazarian J Davis C Spillane L Blumstein H Schneider S Teaching emergency medicine residents evidence-based critical appraisal skills: a controlled trial Ann Emerg Med 1999 34 2 148 154 10424914 10.1016/s0196-0644(99)70222-2

[B32] Bennett KJ Sackett DL Haynes RB Neufeld VR Tugwell P Roberts R A controlled trial of teaching critical appraisal of the clinical literature to medical students J Am Med Asscoc 1987 257 2451 2454 3573243

[B33] Bradley DR Rana GK Martin PW Schumacher RE Real-time, evidence-based medicine instruction: a randomized controlled trial in a neonatal intensive care unit J Med Libr Assoc 2002 90 2 194 201 11999177 PMC100764

[B34] Bradley P Oterholt C Herrin J Nordheim L Bjorndal A Comparison of directed and self-directed learning in evidence-based medicine: a randomised controlled trial Med Educ 2005 39 10 1027 1035 16178830 10.1111/j.1365-2929.2005.02268.x

[B35] Cabell CH Schardt C Sanders L Corey GR Keitz SA Resident utilization of information technology J Gen Intern Med 2001 16 12 838 844 11903763 10.1111/j.1525-1497.2001.10239.x PMC1495306

[B36] Carlock D Anderson J Teaching and assessing the database searching skills of student nurses Nurse Educ 2007 32 6 251 255 17998852 10.1097/01.NNE.0000299477.57185.ba

[B37] Cheng GY Educational workshop improved information-seeking skills, knowledge, attitudes and the search outcome of hospital clinicians: a randomised controlled trial Health Info Libr J 2003 1 22 33 12757433 10.1046/j.1365-2532.20.s1.5.x

[B38] Davis J Crabb S Rogers E Zamora J Khan K Computer-based teaching is as good as face to face lecture-based teaching of evidence based medicine: a randomized controlled trial Med Teach 2008 30 3 302 307 18484458 10.1080/01421590701784349

[B39] Edwards R White M Gray J Fischbacher C Use of a journal club and letter-writing exercise to teach critical appraisal to medical undergraduates Med Educ 2001 35 7 691 694 11437973 10.1046/j.1365-2923.2001.00972.x

[B40] Erickson S Warner ER The impact of an individual tutorial session on MEDLINE use among obstetrics and gynaecology residents in an academic training programme: a randomized trial Med Educ 1998 32 3 269 273 9743780 10.1046/j.1365-2923.1998.00229.x

[B41] Feldstein DA Maenner MJ Srisurichan R Roach MA Vogelman BS Evidence-based medicine training during residency: a randomized controlled trial of efficacy BMC Med Educ 2010 10 59 20807453 10.1186/1472-6920-10-59 PMC2940785

[B42] Forsetlund L Bradley P Forsen L Nordheim L Jamtvedt G Bjorndal A Randomised controlled trial of a theoretically grounded tailored intervention to diffuse evidence-based public health practice [ISRCTN23257060] BMC Med Educ 2003 3 2 12694632 10.1186/1472-6920-3-2 PMC153535

[B43] Fu C Hodges B Regehr G Goldbloom D Garfinkel P Is a journal club effective for teaching critical appraisal skills? Acad Psychiatry 1999 23 205 209

[B44] Gagnon MP Legare F Labrecque M Fremont P Cauchon M Desmartis M Perceived barriers to completing an e-learning program on evidence-based medicine Informat Prim Care 2007 15 2 83 91 10.14236/jhi.v15i2.646 17877870

[B45] Gardois P Calabrese R Colombi N Deplano A Lingua C Longo F Villanacci MC Miniero R Piga A Effectiveness of bibliographic searches performed by paediatric residents and interns assisted by librarians. A randomised controlled trial Health Inform Lib J 2011 28 4 273 284 10.1111/j.1471-1842.2011.00957.x 22051126

[B46] Gehlbach SH Farrow SC Fowkes FG West RR Roberts CJ Epidemiology for medical students: a controlled trial of three teaching methods Int J Epidemiol 1985 14 1 178 181 3988433 10.1093/ije/14.1.178

[B47] Ghali WA Saitz R Eskew AH Gupta M Quan H Hershman WY Ghali WA Successful teaching in evidence-based medicine Med Educ 2000 34 1 18 22 10607274 10.1046/j.1365-2923.2000.00402.x

[B48] Green ML Ellis PJ Impact of an evidence-based medicine curriculum based on adult learning theory J Gen Intern Med 1997 12 12 742 750 9436893 10.1046/j.1525-1497.1997.07159.x PMC1497200

[B49] Griffin NL Schumm RW Instructing occupational therapy students in information retrieval Am J Occup Ther 1992 46 2 158 161 1595827 10.5014/ajot.46.2.158

[B50] Gruppen LD Rana GK Arndt TS A controlled comparison study of the efficacy of training medical students in evidence-based medicine literature searching skills Acad Med 2005 80 10 940 944 16186614 10.1097/00001888-200510000-00014

[B51] Hadley J Kulier R Zamora J Coppus S Weinbrenner S Meyerrose B Decsi T Horvath AR Nagy E Emparanza JI Arvanitis TN Burls A Cabello JB Kaczor M Zanrei G Pierer K Kunz R Wilkie V Wall D Mol BWJ Khan KS Effectiveness of an e-learning course in evidence-based medicine for foundation (internship) training J R Soc Med 2010 103 7 288 294 20522698 10.1258/jrsm.2010.100036 PMC2895523

[B52] Haynes RB Johnston ME McKibbon KA Walker CJ Willan AR A program to enhance clinical use of MEDLINE. A randomized controlled trial Online J Curr Clin Trials 1993 56 [4005 words; 39 paragraphs] 8306006

[B53] Heller RF Peach H Evaluation of a new course to teach the principles and clinical applications of epidemiology to medical students Int J Epidemiol 1984 13 4 533 537 6519896 10.1093/ije/13.4.533

[B54] Hugenholtz NIR Schaafsma FG Nieuwenhuijsen K van Dijk FJH Effect of an EBM course in combination with case method learning sessions: an RCT on professional performance, job satisfaction, and self-efficacy of occupational physicians Int Arch Occup Environ Health 2008 82 1 107 115 18386046 10.1007/s00420-008-0315-3 PMC2467503

[B55] Jalali-Nia S Salsali M Dehghan-Nayeri N Ebadi A Effect of evidence-based education on Iranian nursing students' knowledge and attitude Nurs Health Sci 2011 13 2 221 227 21595816 10.1111/j.1442-2018.2011.00603.x

[B56] Johnston JM Schooling CM Leung GM A randomised-controlled trial of two educational modes for undergraduate evidence-based medicine learning in Asia BMC Med Educ 2009 9 63 19785777 10.1186/1472-6920-9-63 PMC2761870

[B57] Kim SC Brown CE Fields W Stichler JF Evidence-based practice-focused interactive teaching strategy: a controlled study J Adv Nurs 2009 65 6 1218 1227 19445064 10.1111/j.1365-2648.2009.04975.x

[B58] Kim S Willett LR Murphy DJ O'Rourke K Sharma R Shea JA Impact of an evidence-based medicine curriculum on resident Use of electronic resources: a randomized controlled study J Gen Intern Med 2008 23 11 1804 1808 18769979 10.1007/s11606-008-0766-y PMC2585665

[B59] Kitchens J Pfeiffer MP Teaching residents to read the medical literature: a controlled trial of a curriculum in critical appraisal/clinical epidemiology J Gen Intern Med 1989 4 384 387 2795262 10.1007/BF02599686

[B60] Krueger PM Teaching critical appraisal: a pilot randomized controlled outcomes trial in undergraduate osteopathic medical education J Am Osteopath Assoc 2006 106 11 658 662 17192453

[B61] Kulier R Coppus S Zamora J Hadley J Malick S Das K Weinbrenner S Meyerrose B Decsi T Horvath AR Nagy E Emparanza JI Arvanitis TN Burls A Cabello JB Kaczor M Zanrei G Pierer K Stawiarz K Kunz R Mol BWJ Khan KS The effectiveness of a clinically integrated e-learning course in evidence-based medicine: A cluster randomised controlled trial BMC Med Educ 2009 9 21 19435520 10.1186/1472-6920-9-21 PMC2688004

[B62] Landry FJ Pangaro L Kroenke K Lucey C Herbers J A controlled trial of a seminar to improve medical student attitudes toward, knowledge about, and use of the medical literature J Gen Intern Med 1994 9 8 436 439 7965237 10.1007/BF02599058

[B63] Linzer M Brown J Frazier L DeLong E Siegel W Impact of a medical journal club on house-staff reading habits, knowledge, and critical-appraisal skills - a randomized control trial JAMA 1988 260 17 2537 2541 3050179

[B64] MacAuley D McCrum E Brown C Randomised controlled trial of the READER method of critical appraisal in general practice BMJ 1998 316 7138 1134 1137 9552953 10.1136/bmj.316.7138.1134 PMC28517

[B65] MacAuley D McCrum E Critical appraisal using the READER method: a workshop-based controlled trial Fam Pract 1999 16 1 90 93 10321403 10.1093/fampra/16.1.90

[B66] MacRae MacRae HM Regehr G McKenzie M Henteleff H Taylor M Barkun J Fitzgerald W Hill A Richard C Webber E McLeod RS Teaching practicing surgeon’s critical appraisal skills with an internet-based journal club: A randomized, controlled trial Surgery 2004 136 641 646 15349113 10.1016/j.surg.2004.02.003

[B67] Major-Kincade TL Tyson JE Kennedy KA Training pediatric house staff in evidence-based ethics: an exploratory controlled trial J Perinatol 2001 21 3 161 166 11503102 10.1038/sj.jp.7200570

[B68] Martin SD Teaching evidence-based practice to undergraduate nursing students: overcoming obstacles J Coll Teaching Lear 2007 4 1 103 106

[B69] McLeod RS MacRae HM McKenzie ME Victor JC Brasel KJ Evidence based reviews in surgery steering committee. A moderated journal club is more effective than an internet journal club in teaching critical appraisal skills: results of a multicenter randomized controlled trial J Am Coll Surg 2010 211 769 776 21036071 10.1016/j.jamcollsurg.2010.08.016

[B70] Reiter HI Neville AJ Norman G Medline for medical students? Searching for the right answer Adv Health Sci Educ Theory Pract 2000 5 3 221 232 12386464 10.1023/A:1009877514060

[B71] Ross R Verdieck A Introducing an evidence-based medicine curriculum into a family practice residency–is it effective? Acad Med 2003 78 4 412 417 12691976 10.1097/00001888-200304000-00019

[B72] Sánchez-Mendiola M Evidence-based medicine teaching in the Mexican army medical school Med Teach 2004 26 7 661 663 15763862 10.1080/01421590412331282309

[B73] Schaafsma F Hulshof C de Boer A van Dijk F Effectiveness and efficiency of a literature search strategy to answer questions on the etiology of occupational diseases: a controlled trial Int Arch Occup Environ Health 2007 80 3 239 247 16944192 10.1007/s00420-006-0126-3

[B74] Schardt C Adams MB Owens T Keitz S Fontelo P Utilization of the PICO framework to improve searching PubMed for clinical questions BMC Med Inform Decis Mak 2007 7 16 17573961 10.1186/1472-6947-7-16 PMC1904193

[B75] Seelig C Changes over time in the knowledge acquisition practices of internists South Med J 1993 86 7 780 783 8322087 10.1097/00007611-199307000-00013

[B76] Shorten A Wallace MC Crookes PA Developing information literacy: a key to evidence-based nursing Int Nurs Rev 2001 48 2 86 92 11407467 10.1046/j.1466-7657.2001.00045.x

[B77] Shuval K Berkovits E Netzer D Hekselman I Linn S Brezis M Reis S Evaluating the impact of an evidence-based medicine educational intervention on primary care doctors' attitudes, knowledge and clinical behaviour: a controlled trial and before and after study J Eval Clin Pract 2007 13 581 598 17683300 10.1111/j.1365-2753.2007.00859.x

[B78] Smith CA Ganschow PS Reilly BM Evans AT McNutt RA Osei A Saquib M Surabhi S Yadav S Teaching residents evidence-based medicine skills: a controlled trial of effectiveness and assessment of durability J Gen Intern Med 2000 15 10 710 715 11089714 10.1046/j.1525-1497.2000.91026.x PMC1495601

[B79] Stark R Helenius IM Schimming LM Takahara N Kronish I Korenstein D Real-time EBM: from bed board to keyboard and back J Gen Intern Med 2007 22 12 1656 1660 17922170 10.1007/s11606-007-0387-x PMC2219829

[B80] Stevenson K Lewis M Hay E Do physiotherapists' attitudes towards evidence-based practice change as a result of an evidence-based educational programme? J Eval Clin Pract 2004 10 207 217 15189387 10.1111/j.1365-2753.2003.00479.x

[B81] Stevermer JJ Chambliss ML Hoekzema GS Distilling the literature: a randomized, controlled trial testing an intervention to improve selection of medical articles for reading Acad Med 1999 74 70 72 9934299 10.1097/00001888-199901000-00021

[B82] Taylor RS Reeves BC Ewings PE Taylor RJ Critical appraisal skills training for health care professionals: a randomized controlled trial [ISRCTN46272378] BMC Med Educ 2004 4 1 30 15585061 10.1186/1472-6920-4-30 PMC539272

[B83] Thomas KG Thomas MR York EB Dupras DM Schultz HJ Kolars JC Teaching evidence-based medicine to internal medicine residents: the efficacy of conferences versus small-group discussion Teach Learn Med 2005 17 2 130 135 15833722 10.1207/s15328015tlm1702_6

[B84] Verhoeven A Boerma E Meyboom-de Jong B Which literature retrieval method is most effective for GPs? Fam Pract 2000 17 1 30 35 10673485 10.1093/fampra/17.1.30

[B85] Villanueva EV Burrows EA Fennessy PA Rajendran M Anderson JN Improving question formulation for use in evidence appraisal in a tertiary care setting: a randomised controlled trial [ISRCTN66375463 BMC Med Inform Decis Mak 2001 1 4 11716797 10.1186/1472-6947-1-4 PMC59901

[B86] Wallen GR Mitchell SA Melnyk B Fineout-Overholt E Miller-Davis C Yates J Hastings C Implementing evidence-based practice: effectiveness of a structured multifaceted mentorship programme J Adv Nurs 2010 66 12 2761 2771 20825512 10.1111/j.1365-2648.2010.05442.x PMC2981621

[B87] Webber M Currin L Groves N Hay D Fernando N Social workers Can e-learn: evaluation of a pilot post-qualifying e-learning course in research methods and critical appraisal skills for social workers Soc Work Educ 2010 29 1 48 66

[B88] Moher D Schulz K Altman D The CONSORT statement: revised recommendations for improving the quality of reports of parallel group randomized trials BMC Med Res Methodol 2001 1 1 2 11336663 10.1186/1471-2288-1-2 PMC32201

[B89] Portney L Watkins M Foundations of Clinical Research Applications to Practice 2012 3 New Jersey: Prentice-Hall

[B90] Sackett D Strauss S Richardson W Rosenburg W Haynes R Richardson W Evidence-Based Medicine: How to Practice and Teach EBM 1996 1 Edinburgh: Churchill Livingstone

[B91] Evidence-Based Medicine Working Group Evidence-based medicine. A new approach to teaching the practice of medicine JAMA 1992 268 17 2420 2425 1404801 10.1001/jama.1992.03490170092032

[B92] Dean ME Coulter MK Fisher P Jobst K Walach H Delphi Panel of the CONSORT Group Reporting data on homeopathic treatments (RedHot): a supplement to CONSORT Forsch Komplementmed 2006 13 6 368 371 17200612 10.1159/000097073

[B93] Gagnier JJ Boon H Rochon P Moher D Barnes J Bombardier C CONSORT Group Reporting randomized, controlled trials of herbal interventions: an elaborated CONSORT statement Ann Intern Med 2006 144 5 364 367 16520478 10.7326/0003-4819-144-5-200603070-00013

[B94] Boutron I Moher D Altman DG Schulz KF Ravaud P CONSORT Group Extending the CONSORT statement to randomized trials of nonpharmacologic treatment: explanation and elaboration Ann Intern Med 2008 148 4 295 309 18283207 10.7326/0003-4819-148-4-200802190-00008

[B95] MacPherson H Altman DG Hammerschlag R Youping L Taixiang W White A Moher D STRICTA Revision Group Revised STandards for Reporting Interventions in Clinical Trials of Acupuncture (STRICTA): extending the CONSORT statement PLoS Med 2010 7 6 e1000261 20543992 10.1371/journal.pmed.1000261 PMC2882429

[B96] Eysenbach G CONSORT-EHEALTH Group CONSORT-EHEALTH: improving and standardizing evaluation reports of Web-based and mobile health interventions J Med Internet Res 2011 13 4 e126 22209829 10.2196/jmir.1923 PMC3278112

[B97] Harrington NG Noar SM Reporting standards for studies of tailored interventions Health Educ Res 2012 27 2 331 342 22156230 10.1093/her/cyr108

