# Supplementary material for: A systematic review of how studies describe educational interventions for evidence-based practice: stage 1 of the development of a reporting guideline
Source: BMC Med Educ. 2014 Jul 24;14:152. doi: 10.1186/1472-6920-14-152 (PMC4113129; doi:10.1186/1472-6920-14-152)
Supplement: Additional file 1 — MEDLINE Search strategy for the OVID interface. The MEDLINE search strategy we used for the systematic review using the OVID interface. [file 1472-6920-14-152-S1.pdf]

Additional file 1 for manuscript

A systematic review of how studies describe educational interventions for evidence-based practice: Stage 1 of the development of a reporting guideline.

Additional file 1.MEDLINE Search strategy for OVID interface.

**Search Terms**	***mp = key word exp = explode function used**
**1**	exp medicine
**2**	medic*.mp
**3**	exp nursing
**4**	exp nurses
**5**	nurs*.mp
**6**	exp allied health occupations
**7**	exp health occupations
**8**	exp health personnel
**9**	“allied health”.mp
**10**	physiotherap*.mp
**11**	“physical therap*”.mp
**12**	exp “physical therapy (specialty)”
**13**	exp occupational therapy
**14**	“occupational therapy”.mp
**15**	exp speech therapy
**16**	“speech therapy”.mp
**17**	“speech pathology”.mp
**18**	diet*.mp
**19**	exp dietetics
**20**	nutrit*.mp
**21**	exp social work
**22**	“social work*”.mp
**23**	exp psychology
**24**	psycholog*.mp
**25**	exp podiatry
**26**	podiatr*.mp
**27**	“ambulance paramedic”.mp
**28**	“ambulance officer”.mp
**29**	exp music therapy
**30**	“music therapy”.mp
**31**	exp art therapy
**32**	“art therap*”.mp
**33**	exp osteopathic medicine
**34**	osteopath*.mp
**35**	exp chiropractic
**36**	chiropractor*.mp
**37**	exp dentists
**38**	exp dentistry
**39**	dentist*.mp
**40**	exp optometry
**41**	optometry*.mp

**42**	optometrist*.mp
**43**	“medical radiation*”.mp
**44**	“medical radiation science”.mp
**45**	exp radiography
**46**	exp radiology
**47**	exppharmac*
**48**	pharmac*.mp
**49**	exp pharmacists
**50**	“exercise physiology*”.mp
**51**	or/1-50
**52**	instrument*.mp
**53**	exp questionnaires
**54**	questionnair*.mp
**55**	survey*.mp
**56**	tool*.mp
**57**	exp data collection
**58**	or/52-57
**59**	EBP.mp.
**60**	exp evidence based practice
**61**	EBM.mp
**62**	exp evidence based medicine
**63**	BEME.mp
**64**	“best evidence medical education”.mp
**65**	“research evidence”.mp
**66**	“evidence based health care”.mp
**67**	exp evidence based emergency medicine
**68**	exp evidence based nursing
**69**	exp evidence based dentistry
**70**	“critical appraisal”.mp
**71**	or/ 59-70
**72**	teach*.mp
**73**	exp teaching
**74**	exp learning
**75**	learn*. mp
**76**	course*.mp
**77**	exp curriculum
**78**	curriculum*.mp
**79**	train*.mp
**80**	exp education
**81**	eduact*.mp
**82**	exp program evaluation
**83**	exp program development
**84**	exp professional competence
**85**	“journal club*”.mp
**86**	workshop*.mp
**87**	exp students
**88**	student*.mp
**89**	or/ 72 -88
**90**	(51 and 58 and 71 and 89)
**91**	Limit 90 to (adults >19 years
