# Supplementary material for: A systematic review of how studies describe educational interventions for evidence-based practice: stage 1 of the development of a reporting guideline
Source: BMC Med Educ. 2014 Jul 24;14:152. doi: 10.1186/1472-6920-14-152 (PMC4113129; doi:10.1186/1472-6920-14-152)
Supplement: Additional file 2 — Summary of data items reported for included studies. A summary of the reporting for the 25 data extraction items across the studies (n = 61) included in the review. [file 1472-6920-14-152-S2.pdf]

Additional file 2 Summary of data items reported for included studies (n=61)

Source (First author, year, ref no)			Learners	Instructors			Intervention										
			Adherence or attendance	Number	Profession	Experience	Educational framework	Teaching strategies	Materials	Number of sessions	Duration of sessions	Frequency of sessions	Session setting	Program duration	EBP components OR "Steps of EBP"	Reference for EBP content	Confounding issues
Fritsche,L.	2002	6	Y	Y	N	Y	N	Y	N	Y	Y	Y	N	Y	N	N	Y
Akl,E.	2004	22	Y	N	Y	N	Y	Y	Y	Y	N	Y	Y	Y	N	Y	Y
Arlt,S.	2011	23	Y	N	N	N	N	Y	Y	Y	Y	Y	Y	Y	N	N	Y
Badgett,R.	2001	24	N	N	N	N	N	Y	Y	Y	Y	N	Y	N	N	N	Y
Bazarian,J.	1999	25	Y	N	Y	N	Y	Y	Y	Y	Y	Y	Y	Y	N	N	Y
Bennett,K.	1987	26	N	Y	Y	N	Y	Y	Y	Y	Y	Y	Y	Y	N	N	Y
Bradley,DR.	2002	27	N	Y	Y	N	Y	Y	Y	Y	N	Y	Y	Y	N	N	Y
Bradley,P.	2005	28	Y	N	N	N	Y	Y	Y	Y	Y	Y	Y	Y	Y	Y	Y
Cabell,C.	2001	29	N	N	Y	N	N	Y	Y	Y	Y	Y	Y	Y	Y	N	Y
Carlock,D.	2007	30	Y	Y	Y	N	N	Y	N	Y	Y	Y	Y	Y	N	N	N
Cheng,G.	2003	31	Y	N	N	N	Y	Y	N	Y	Y	Y	Y	Y	N	N	Y
Davis,J.	2008	32	Y	Y	N	N	Y	Y	Y	Y	Y	Y	Y	Y	N	N	Y
Edwards,R.	2001	33	N	N	N	N	N	Y	Y	Y	Y	N	Y	N	N	N	Y
Erickson,S.	1998	34	Y	Y	Y	N	N	Y	N	Y	Y	Y	Y	Y	N	N	Y
Feldstein,D.	2010	35	N	Y	Y	N	N	Y	Y	Y	Y	Y	Y	Y	Y	N	Y
Forsetlund,L.	2003	36	Y	N	N	N	Y	Y	Y	Y	N	N	N	Y	N	N	Y
Fu,C.	1999	37	Y	Y	Y	N	N	Y	Y	Y	Y	Y	Y	Y	N	N	Y
Gagnon,M.	2007	38	Y	N	NA	N	Y	Y	Y	Y	N	N	N	Y	N	N	Y
Gardois,P.	2011	39	Y	Y	Y	Y	N	Y	Y	Y	Y	Y	Y	Y	N	N	Y
Gehlbach,S.	1985	40	N	Y	Y	N	N	Y	Y	Y	Y	Y	Y	Y	N	N	Y
Ghali,W.	2000	41	N	N	N	N	N	Y	Y	Y	Y	Y	Y	Y	Y	Y	Y
Green,M.	1997	42	Y	N	Y	N	Y	Y	Y	Y	Y	Y	Y	Y	Y	N	Y
Griffin,N.	1992	43	N	Y	Y	N	N	Y	Y	Y	Y	Y	Y	Y	N	N	N
Gruppen,L.	2005	44	N	Y	Y	N	N	Y	Y	Y	Y	Y	Y	Y	N	N	Y
Hadley,J.	2010	45	N	Y	N	N	Y	Y	Y	Y	Y	Y	Y	Y	N	Y	Y
Haynes,R.	1993	46	Y	N	Y	Y	N	Y	Y	Y	N	N	Y	Y	N	N	Y
Heller,R.	1984	47	N	N	N	N	N	Y	N	Y	Y	Y	Y	Y	N	N	Y
Hugenholtz,N.	2008	48	Y	N	Y	N	N	Y	Y	Y	Y	Y	Y	Y	N	N	Y

Jalali-Nia, S.	2011	49	N	Y	N	Y	N	Y	N	Y	Y	Y	Y	Y	N	N	Y
Johnston,J.	2009	50	N	N	N	N	Y	Y	Y	Y	Y	N	Y	N	N	N	Y
Kim,S.	2008	51	N	Y	Y	N	N	Y	Y	Y	Y	Y	Y	Y	N	N	Y
Kim,S.	2009	52	N	N	N	N	Y	Y	N	N	N	N	Y	Y	Y	N	Y
Kitchens, J.	1989	53	N	N	N	N	N	Y	Y	Y	Y	Y	Y	Y	N	N	Y
Krueger,P.	2006	54	N	Y	Y	N	N	Y	Y	Y	N	Y	Y	Y	N	Y	Y
Kulier,R.	2009	55	N	N	N	N	Y	Y	Y	N	N	N	Y	Y	N	Y	Y
Landry,F.	1994	56	N	N	N	N	N	Y	Y	Y	Y	Y	N	Y	N	N	Y
Linzer,M.	1988	57	Y	N	Y	N	N	Y	Y	Y	N	N	Y	N	N	N	Y
MacAuley,D.	1998	58	N	N	NA	N	N	Y	Y	N	N	N	N	N	N	N	Y
MacAuley,D.	1999	59	N	N	N	N	N	N	N	Y	Y	Y	Y	Y	N	N	Y
MacRae,H.	2004	60	Y	Y	N	Y	Y	Y	Y	Y	N	Y	Y	Y	N	N	Y
Major-Kincade, T.	2001	61	N	Y	Y	N	N	Y	N	Y	Y	Y	Y	Y	N	Y	Y
Martin,S.	2007	62	N	Y	N	N	N	Y	N	N	N	N	N	N	N	N	Y
McLeod,R.	2010	63	Y	N	Y	N	N	Y	Y	Y	N	Y	Y	Y	N	Y	Y
Reiter,H.	2000	64	N	N	NA	N	Y	Y	Y	Y	N	Y	Y	Y	N	N	Y
Ross,R.	2003	65	N	N	NA	N	N	Y	Y	Y	Y	Y	Y	Y	N	N	Y
Sanchez-Mendiola,M.	2004	66	N	N	N	N	N	Y	Y	Y	Y	N	N	N	N	Y	Y
Schaafsma,F.	2007	67	N	N	N	N	N	N	Y	N	Y	N	Y	Y	Y	N	Y
Schardt,C.	2007	68	N	N	N	N	N	Y	Y	N	N	N	N	N	N	Y	Y
Seelig,C. B.	1993	69	N	N	N	N	Y	Y	N	Y	Y	Y	N	Y	N	N	Y
Shorten,A.	2001	70	N	N	Y	N	Y	Y	Y	N	N	N	Y	N	N	Y	N
Shuval,K.	2007	71	Y	Y	Y	Y	Y	Y	N	Y	N	Y	Y	Y	Y	Y	Y
Smith,C.	2000	72	Y	Y	Y	N	N	Y	N	Y	Y	Y	Y	Y	N	Y	Y
Stark,R.	2007	73	Y	Y	Y	Y	N	Y	Y	Y	Y	Y	Y	Y	N	Y	Y
Stevenson,K.	2004	74	N	N	Y	N	Y	Y	N	Y	Y	N	N	Y	N	Y	Y
Stevermer,J.	1999	75	N	N	N	N	Y	Y	Y	Y	Y	Y	Y	Y	N	N	N
Taylor,R.	2004	76	Y	Y	N	Y	Y	Y	Y	Y	Y	Y	N	Y	N	Y	Y
Thomas,K.	2005	77	Y	N	N	N	N	Y	Y	Y	Y	Y	Y	Y	Y	N	Y
Verhoeven,A.	2000	78	N	Y	Y	N	N	Y	Y	Y	Y	Y	Y	Y	N	N	Y
Villanueva,E.	2001	79	N	N	N	N	N	Y	Y	Y	N	N	Y	N	N	Y	Y
Wallen,G.	2010	80	N	N	N	N	N	Y	N	N	N	Y	N	N	N	Y	Y
Webber,M.	2010	81	N	N	N	N	N	Y	Y	Y	Y	Y	Y	Y	N	N	Y

NA=not applicable (e.g. online intervention without instructor)

Reference list for Additional file 2

6. Fritsche L, Greenlagh T, Falck-Ytter Y, Neumayer HH, Kunz R: **Do short courses in evidence based medicine improve knowledge and skills? Validation of Berlin questionnaire and before and after study of courses in evidence based medicine**.*Br Med J* 2002, **325:**1338–1341.

25. Akl EA, Izuchukwu IS, El-Dika S, Fritsche L, Kunz R, Schünemann HJ: **Integrating an evidence-based medicine rotation into an internal medicine residency program.** *Academic Medicine* 2004, **79**: 897-904.
26. Arlt SP, Heuwieser W: **Training students to appraise the quality of scientific literature.** *Journal of Veterinary Medical Education* 2011, **38**:135-140.
27. Badgett RG, Paukert JL, Levy LS: **Teaching clinical informatics to third-year medical students: Negative results from two controlled trials.** *BMC Med Educ* 2001, **1**:3.
28. Bazarian J, Davis C, Spillane L, Blumstein H, Schneider S: **Teaching emergency medicine residents evidence-based critical appraisal skills: A controlled trial**. *Ann Emerg Med* 1999, **34**:148-154.
29. Bennett KJ, Sackett DL, Haynes RB, Neufeld VR, Tugwell P, Roberts R: **A controlled trial of teaching critical appraisal of the clinical literature to medical students.** *J Am Med Asscoc* 1987, **257**:2451-2454.
30. Bradley DR, Rana GK, Martin PW, Schumacher RE: **Real-time, evidence-based medicine instruction: A randomized controlled trial in a neonatal intensive care unit**. *J Med Libr Assoc* 2002, **90**:194-201.

31. Bradley P, Oterholt C, Herrin J, Nordheim L, Bjorndal A: **Comparison of directed and self-directed learning in evidence-based medicine: A randomised controlled trial**. *Med Educ* 2005, **39:**1027-1035.

32. Cabell CH, Schardt C, Sanders L, Corey GR, Keitz SA: **Resident utilization of information technology.** *J Gen Intern Med* 2001, **16:**838-844.

33. Carlock D, Anderson J: **Teaching and assessing the database searching skills of student nurses.** *Nurse Educ* 2007, **32**:251-255.

34. Cheng GY: **Educational workshop improved information-seeking skills, knowledge, attitudes and the search outcome of hospital clinicians: A randomised controlled trial.** *Health Info Libr J* 2003, **1:**22-33.

35. Davis J, Crabb S, Rogers E, Zamora J, Khan K: **Computer-based teaching is as good as face to face lecture-based teaching of evidence based medicine: A randomized controlled trial.** *Med Teach* 2008, **30:**302-307.

36. Edwards R, White M, Gray J, Fischbacher C: **Use of a journal club and letter-writing exercise to teach critical appraisal to medical undergraduates**. *Med Educ* 2001, **35:**691-694.

37. Erickson S, Warner ER: **The impact of an individual tutorial session on MEDLINE use among obstetrics and gynaecology residents in an academic training programme: A randomized trial.** *Med Educ* 1998, **32:**269-273.

38. Feldstein DA, Maenner MJ, Srisurichan R, Roach MA, Vogelman BS: **Evidence-based medicine training during residency: A randomized controlled trial of efficacy.** *BMC Med Educ* 2010, **10:**59.

39. Forsetlund L, Bradley P, Forsen L, Nordheim L, Jamtvedt G, Bjorndal A: **Randomised controlled trial of a theoretically grounded tailored intervention to diffuse evidence-based public health practice [ISRCTN23257060].** *BMC Med Educ* 2003, **3:**2.
40. Fu C, Hodges B, Regehr G, Goldbloom D, Garfinkel P: **Is a journal club effective for teaching critical appraisal skills? A controlled trial with residents in psychiatry**. *Academic Psychiatry* 1999, **23:**205-209.
41. Gagnon MP, Legare F, Labrecque M, Fremont P, Cauchon M, Desmartis M: **Perceived barriers to completing an e-learning program on evidence-based medicine.** *Informatics in Primary Care* 2007, **15:**83-91.
42. Gardois P, Calabrese R, Colombi N, Deplano A, Lingua C, Longo F, Villanacci MC, Miniero R, Piga A: **Effectiveness of bibliographic searches performed by paediatric residents and interns assisted by librarians. A randomised controlled trial**. *Health Information and Libraries Journal* 2011, **28:**273-284.
43. Gehlbach SH, Farrow SC, Fowkes FG, West RR, Roberts CJ: **Epidemiology for medical students: A controlled trial of three teaching methods.** *International Journal of Epidemiology* 1985, **14:**178-181.
44. Ghali WA, Saitz R, Eskew AH, Gupta M, Quan H, Hershman WY: **Successful teaching in evidence-based medicine.** *Med Educ* 2000, **34:**18-22.
45. Green ML, Ellis PJ**: Impact of an evidence-based medicine curriculum based on adult learning theory.** *Journal of General Internal Medicine* 1997, **12**:742-750.
46. Griffin NL, Schumm RW: **Instructing occupational therapy students in information retrieval.** *Am J OccupTher* 1992, **46**:158-161.

47. Gruppen LD, Rana GK, Arndt TS: **A controlled comparison study of the efficacy of training medical students in evidence-based medicine literature searching skills**. *Acad Med* 2005, **80:**940-944.

48. Hadley J, Kulier R, Zamora J, Coppus SF, Weinbrenner S, Meyerrose B, Decsi T, Horvath AR, Nagy E, Emparanza JI, Arvanitis TN, Burls A, Cabello JB, Kaczor M, Zanrei G, Pierer K, Kunz R, Wilkie V, Wall D, Mol BJ, Khan KS: **Effectiveness of an e-learning course in evidence-based medicine for foundation (internship) training.** *Journal of the Royal Society of Medicine* 2010, **103:**288-294.

49. Haynes RB, Johnston ME, McKibbon KA, Walker CJ, Willan AR: **A program to enhance clinical use of MEDLINE. A randomized controlled trial.** *Online J Curr Clin Trials* 1993, **11:**56.

50. Heller RF, Peach H: **Evaluation of a new course to teach the principles and clinical applications of epidemiology to medical students.** *Int J Epidemiol* 1984, **13:**533-537.

51. Hugenholtz NI, Schaafsma FG, Nieuwenhuijsen K, van Dijk FJ: **Effect of an EBM course in combination with case method learning sessions: An RCT on professional performance, job satisfaction, and self-efficacy of occupational physicians.** *International Archives of Occupational & Environmental Health* 2008, **82:**107-115.

52. Jalali-Nia S, Salsali M, Dehghan-Nayeri N, Ebadi A: **Effect of evidence-based education on iranian nursing students' knowledge and attitude.** *Nurs Health Sci* 2011, **13:**221-227.

53. Johnston JM, Schooling CM, Leung GM: **A randomised-controlled trial of two educational modes for undergraduate evidence-based medicine learning in Asia**. *BMC Medical Educ* 2009, **9:**63.

54. Kim S, Willett LR, Murphy DJ, O'Rourke K, Sharma R, Shea JA: **Impact of an evidence-based medicine curriculum on resident use of electronic resources: A randomized controlled study.** *Journal of General Internal Medicine* 2008, **23:**1804-1808.

55. Kim SC, Brown CE, Fields W, Stichler JF: **Evidence-based practice-focused interactive teaching strategy: A controlled study**. *J Adv Nurs* 2009, **65:**1218-1227.

56. Kitchens J, Pfeiffer MP: **Teaching residents to read the medical literature: A controlled trial of a curriculum in critical appraisal /clinical epidemiology.** *Journal of General Internal Medicine* 1989, **4:**384-387.

57. Krueger PM: **Teaching critical appraisal: A pilot randomized controlled outcomes trial in undergraduate osteopathic medical education**. *J Am Osteopath Assoc* 2006, **106:**658-662.

58. Kulier R, Coppus S, Zamora J, Hadley J, Malick S, Das K, Weinbrenner S, Meyerrose B, Decsi T, Horvath AR, Nagy E, Emparanza JI, Arvanitis TN, Burls A, Cabello J, Kaczor M, Zanrei G, Peirer K, Stawiarz K, Kunz R, Mol B, Khan KS: **The effectiveness of a clinically integrated e-learning course in evidence-based medicine: A cluster randomised controlled trial.** *BMC Med Educ* 2009, **9**:21.

59. Landry FJ, Pangaro L, Kroenke K, Lucey C, Herbers J: **A controlled trial of a seminar to improve medical student attitudes toward, knowledge about, and use of the medical literature.** *Journal of General Internal Medicine* 1994, **9:**436-439.

60. Linzer M, Brown J, Frazier L, Delong E, Siegel W: **Impact of a medical journal club on house-staff reading habits, knowledge, and critical-appraisal skills - a randomized control trial***. J Am Med Assoc* 1988, **260:**2537-2541.

61. MacAuley D, McCrum E, Brown C: **Randomised controlled trial of the READER method of critical appraisal in general practice.** *BMJ* 1998, **316:**1134-1137.

62. MacAuley D, McCrum E: **Critical appraisal using the READER method: A workshop-based controlled trial.** *Fam Pract* 1999, **16:** 90-93.
63. MacRae HM, Regehr G, McKenzie M, Henteleff H, Taylor M, Barkun J, Fitzgerald W, Hill A, Richard C, Webber E, McLeod RS: **Teaching practicing surgeon's critical appraisal skills with an internet-based journal club: A randomized, controlled trial**. *Surgery* 2004, **136:** 641-646.
64. Major-Kincade TL, Tyson JE, Kennedy KA: **Training pediatric house staff in evidence-based ethics: An exploratory controlled trial**. *J Perinatol* 2001, **21:**161-166.
65. Martin SD: **Teaching evidence-based practice to undergraduate nursing students: Overcoming obstacles**. *Journal of College Teaching & Learning* 2007, **4:**103-106.
66. McLeod RS, MacRae HM, McKenzie ME, Victor JC, Brasel KJ: **Evidence Based Reviews in Surgery Steering Committee. A moderated journal club is more effective than an internet journal club in teaching critical appraisal skills: Results of a multicenter randomized controlled trial**. *J Am Coll Surg* 2010, **211:**769-776.
67. Reiter HI, Neville AJ, Norman G: **Medline for medical students? Searching for the right answer.** *Adv Health Sci Educ Theory Pract* 2000, **5:**221-232.
68. Ross R, Verdieck A: **Introducing an evidence-based medicine curriculum into a family practice residency-is it effective?** *Acad Med* 2003, **78:**412-417.
69. Sánchez-Mendiola M: **Evidence-based medicine teaching in the mexican army medical school**. *Med Teach* 2004, **26:**661-663.
70. Schaafsma F, Hulshof C, de Boer A, van Dijk F: **Effectiveness and efficiency of a literature search strategy to answer questions on the etiology of occupational diseases: A controlled trial.** *Int Arch Occup Environ Health* 2007, **80:**239-247.

71. Schardt C, Adams MB, Owens T, Keitz S, Fontelo P: **Utilization of the PICO framework to improve searching PubMed for clinical questions.** *BMC Med Inform Decis Mak* 2007, **7:**16.

72. Seelig CB: **Changes over time in the knowledge acquisition practices of internists.** *South Med J* 1993, **86:**780-783

73. Shorten A, Wallace MC, Crookes PA: **Developing information literacy: A key to evidence-based nursing.** *Int Nurs Rev* 2001, **48:**86-92.

74. Shuval K, Berkovits E, Netzer D, Hekselman I, Linn S, Brezis M, Reis, S: **Evaluating the impact of an evidence-based medicine educational intervention on primary care doctors' attitudes, knowledge and clinical behaviour: A controlled trial and before and after study.** *J Eval Clin Pract* 2007, **13:**581-598.

75. Smith CA, Ganschow PS, Reilly BM, Evans AT, McNutt RA, Osei A, Saquib M, Surhabi S, Yadav S: **Teaching residents evidence-based medicine skills: A controlled trial of effectiveness and assessment of durability.** *J Gen Intern Med* 2000, **15:**710-715.

76. Stark R, Helenius IM, Schimming LM, Takahara N, Kronish I, Korenstein D: **Real-time EBM: From bedboard to keyboard and back.** *Journal of General Internal Medicine* 2007, **22:**1656-1660.

77. Stevenson K, Lewis M, Hay E: **Do physiotherapists' attitudes towards evidence-based practice change as a result of an evidence-based educational programme?** *J Eval Clin Pract.* 2004, **10:**207-217.

78. Stevermer JJ, Chambliss ML, Hoekzema GS: **Distilling the literature: A randomized, controlled trial testing an intervention to improve selection of medical articles for reading.** *Acad Med* 1999, **74:**70-72.

79. Taylor RS, Reeves BC, Ewings PE, Taylor RJ: **Critical appraisal skills training for health care professionals: A randomized controlled trial [ISRCTN46272378].** *BMC Med Educ* 2004, **4:**30.

80. Thomas KG, Thomas MR, York EB, Dupras DM, Schultz HJ, Kolars JC: **Teaching evidence-based medicine to internal medicine residents: The efficacy of conferences versus small-group discussion.** *Teach Learn Med* 2005, **17:**130-135.

81. Verhoeven A, Boerma E, Meyboom-de Jong B. **Which literature retrieval method is most effective for GPs?** *Fam Pract* 2000, **17:**30-35.

82. Villanueva EV, Burrows EA, Fennessy PA, Rajendran M, Anderson JN: **Improving question formulation for use in evidence appraisal in a tertiary care setting: a randomised controlled trial [ISRCTN66375463].** *BMC Med Inform Decis Mak* 2001, **1:**4.

83. Wallen GR, Mitchell SA, Melnyk B, Fineout-Overholt E, Miller-Davis C, Yates J, Hastings C: **Implementing evidence-based practice: Effectiveness of a structured multifaceted mentorship programme.** *J Adv Nurs* 2010, **66:** 2761-2771.

84. Webber M, Currin L, Groves N, Hay D, Fernando N: **Social workers can e-learn: Evaluation of a pilot post-qualifying e-learning course in research methods and critical appraisal skills for social workers.** *Social Work Education* 2010, **29:**48-66.
