# Supplementary material for: A systematic review of how studies describe educational interventions for evidence-based practice: stage 1 of the development of a reporting guideline
Source: BMC Med Educ. 2014 Jul 24;14:152. doi: 10.1186/1472-6920-14-152 (PMC4113129; doi:10.1186/1472-6920-14-152)
Supplement: Additional file 3 — Confounding items/study limitations allocated using the CONSORT checklist. A summary table of the confounding information items and study limitations reported by included studies which were allocated using the CONSORT checklist items. [file 1472-6920-14-152-S3.pdf]

Additional file3

Confounding items and study limitations allocated using the CONSORT checklist (n=197)

CONSORT section and item number (3-22)		CONSORT Checklist item	No. studies reporting n (%)
**METHODS**			
Trial design	3a	Description of trial design (such as parallel, factorial) including allocation ratio	**3 (2)**
	3b	Important changes to methods after trial commencement (such as eligibility criteria), with reasons	**0**
Participants	4a	Eligibility criteria for participants	**12 (6)**
	4b	Settings and locations where the data were collected	**4 (2)**
Interventions	5	The interventions for each group with sufficient details to allow replications, including how and when they were actually administered	**42 (21)**
Outcomes	6a	Completely pre-defined primary and secondary outcome measures, including how and when they were assessed.	**0**
	6b	Any changes to trial outcomes after the trial commenced, with reasons	**0**
Sample size	7a	How sample size was determined	**27 (14)**
	7b	When applicable, explanation of any interim analyses and stopping guidelines	**0**
Randomisation			**0**
Sequence generation	8a	Method used to generate the random allocation sequence	**0**
	8b	Type of randomisations; details of any restrictions (such as blocking and block size)	**15 (8)**
Allocation concealment mechanism	9	Mechanism used to implement the random allocation sequence (such as sequentially numbered containers), describing any steps taken to conceal the sequence until interventions were assigned.	**0**
Implementation	10	Who generated the random allocation sequence, who enrolled participants, and who assigned participants to interventions	**0**
Blinding	11a	If done, who was blinded after assignment to interventions (for example, participants, care providers, those assessing outcomes) and how	**2 (1)**
	11b	If relevant, description of the similarity of interventions	**1 (1)**
Statistical methods	12a	Statistical methods used to compare groups for primary and secondary outcomes	**0**
	12b	Methods for additional analyses, such as subgroup analyses and adjusted analyses	**0**
**RESULTS**			
Participant flow	13a	For each group, the numbers of participants who were randomly assigned, received intended treatment, and were analysed for the primary outcome	**14 (7)**
	13b	For each group, losses and exclusions after randomisation, together with reasons	**0**
Recruitment	14a	Dates defining the periods of recruitment and follow up	**12 (6)**
	14b	Why the trial ended or was stopped	**0**
Baseline data	15	A table showing baseline demographic and clinical characteristics for each group	**19 (10)**
Numbers analysed	16	For each group, number of participants (denominator) included in each analysis and whether the analysis was by original or assigned groups	**0**
Outcomes and estimation	17a	For each primary and secondary outcome, results for each group, and the estimated effect size and its precision (such as 95% confidence interval)	**26 (13)**
	17b	For binary outcomes, presentation of both absolute and relative effect sizes is recommended	**0**
Ancillary analyses	18	Results of any other analyses performed, including subgroup analyses and adjusted analyses, distinguishing pre-specified from exploratory	**0**
Harms	19	All important harms or unintended effects in each group	**0**
**DISCUSSION**			**10 (5)**
Limitations	20	Trial limitations, addressing sources of potential bias, imprecision, and, if relevant, multiplicity of analyses	**0**
Generalisability	21	Generalisability (external validity, applicability) of the trial findings	**10 (5)**
